# Study on Enhancing the Corrosion Resistance and Photo-Thermal Antibacterial Properties of the Micro-Arc Oxidation Coating Fabricated on Medical Magnesium Alloy

**DOI:** 10.3390/ijms231810708

**Published:** 2022-09-14

**Authors:** Tianlu Li, Yun Zhao, Minfang Chen

**Affiliations:** 1 School of Materials Science and Engineering, Tianjin University of Technology, Tianjin 300384, China; 2 Key Laboratory of Display Materials and Photoelectric Device, Ministry of Education, Tianjin University of Technology, Tianjin 300384, China; 3 National Demonstration Center for Experimental Function Materials Education, Tianjin University of Technology, Tianjin 300384, China

**Keywords:** magnesium alloy, micro-arc oxidation, corrosion resistance, photo-thermal therapy, anti-bacteria

## Abstract

Photo-thermal antibacterial properties have attracted much attention in the biomedical field because of their higher antibacterial efficiency. Through fabricating micro-arc oxidation coatings with different treating current densities set on a Mg-Zn-Ca alloy, the present study tried to systematically investigate and optimize the corrosion resistance and photo-thermal antibacterial properties of MAO coatings. The results indicated that different current densities had great influence on the corrosion resistance and photo-thermal property of the MAO coatings, and a current density at 30 A·dm^−2^ exhibited the best corrosion resistance, light absorption capacity at 808 nm, and photo-thermal capability, simultaneously with good antibacterial activity against *Staphylococcus aureus* (*S. aureus*) and *Escherichia coli* (*E. coli*). This photo-thermal property of MAO coatings was probably related to the effect of current density on MgO content in the coating that could promote the separation of photo-generated electron carriers and hinder the recombination of photo-generated electron carriers and holes.

## 1. Introduction

Compared with traditional metallic biomaterials such as stainless steel and titanium alloys, magnesium alloys have a Young’s modulus that is more similar to the human skeleton, reducing stress shielding during load transferring at the interface between human bone and implant [1]. Moreover, the superior biocompatibility and the natural degradation of Mg alloys in the human environment can effectively solve the lesion of secondary surgery [2], having excellent potential for biodegradable orthopedic applications. However, the defect of magnesium and its alloys is that they are susceptible to corrosion in the human environment. They degrade too quickly and lose mechanical strength prematurely [3]. Therefore, regulating the degradation characteristics of Mg alloys is the key to their wider clinical application.

Implant infection is a common and severe complication in clinical applications [4]. An implant’s surface is conducive to bacterial adhesion, colonization, and biofilm formation because the implant lacks antibacterial properties, which may lead to bacterial infection and the failure of surgery [5].

Due to quick corrosion and bacterial infection in bone implantation, it is necessary to prepare biodegradable and antibacterial surfaces on magnesium alloys. The defense of Mg in anti-corrosion and anti-bacterial properties can usually be acquired by strategies like alloying, composite formation, and surface modification. The surface modification of a magnesium alloy can improve the corrosion resistance and improve specific biological activity and bacteriostasis without changing the mechanical properties, for example, adding metal ions to the coating such as cobalt ions [6], copper ions [7], and silver ions [8]. Metal particles can penetrate bacterial membranes and disrupt life activities inside the bacteria, interfering with their metabolism and killing bacteria. However, metal particles or metal oxides cannot effectively control the release of metal ions because of their sudden release phenomenon. With high cytotoxicity, the prolonged use of metal ions stimulates bacterial tolerance [6]. Considering that antibiotics and other drugs have been the main methods in the clinical environment up to now [9], the researchers tried to make the coatings with drugs give the magnesium and alloy antibacterial properties. However, the application of drugs to magnesium and the alloy surface is confronted with great challenges. It is well known that wound healing requires a specific period. If the drug is released at the beginning of implantation, it will be toxic to the body and is not conducive to inhibiting infection in the late course in the meantime. In addition, the use of antibiotic drugs can lead to a large number of bacteria resistant to antibiotics [10].

In recent years, because of its targeted selectivity, remote controllability, minimal invasiveness, and excellent biosafety [11], photo-thermal therapy (PTT) has aroused more and more research interests. PTT therapy is based on the introduction of a photosensitizing agent, which can convert light into heat energy, thus inducing cell death via hyperthermia. Therefore, a composite coating was constructed on the surface of an alloy with the introduction by hydrothermal or sol-gel methods of a photosensitizing agent to achieve the photo-thermal effect. For example, Zhang et al. [12] used a MoS_2_ and TiO_2_ coating fabricated on Ti by a hybrid process of micro-arc oxidation (MAO) and hydrothermal treatment. The results showed that the composite coating exhibited excellent antibacterial activity under 808 nm NIR light irradiation. Zhang et al. [13] synthesized MoSe_2_/CHI composite coatings on the surface of a TiO_2_ coating prepared by micro-arc oxidation. The results showed that, under 808 nm light irradiation, the TiO_2_/MoSe_2_/CHI composite coating presented good photo-thermal antibacterial properties and promoted bone integration in vivo. 

Liu et al., used a two-step method for the in situ growth of Mg-Fe LDH films [14] and Mg-Mn LDH [15] on the MAO coating in the work. The results showed that the composite coating has good corrosion resistance and good antibacterial properties in vitro under an 808 nm NIR light. Notably, their study showed that the MAO coating on the Mg alloy also has certain photo-thermal properties under 808 nm irradiation. A MAO coating on a magnesium alloy is an effective way to improve the corrosion resistance of the substrate.

At present studies, most of the research on the photo-thermal therapy of alloy coating has been merely conducted through an analysis of the effect of the secondary treatment. But the techniques used above rely on multi-step synthesis, leading to shortages including time-wasting. Meanwhile, the degradation behavior of composite coatings is more complicated. So, it is difficult to fabricate the photo-thermal coating on magnesium alloys in a simple way. Comparing the two photo-thermal composite coatings studied by Zhang et al., the present study found out that the TiO_2_ coating prepared by changing the current parameters had different heating curves after irradiation under an 808 nm NIR (0.6 W·cm^−2^) light for 15 min. It showed that the micro-arc oxidation’s electrical parameters can change the MAO coating’s photo-thermal ability on a titanium alloy due to the good photocatalytic performance of TiO_2_. MgO has received great attention in photocatalysis, because of its sufficient reactive sites and native structural defects. Some researchers proved that MgO plays an important role in the improvement of the photocatalytic activity, such as Liang et al., Peng et al., Wu et al., etc. [16,17,18,19,20,21]. MAO is the application of a voltage on a metal to oxidize the metal surface, and the functional coating, mainly composed of matrix metal oxide, is formed on the surface of the base metal. That is to say, the MAO coating on the Mg alloy surface is composed of a ceramic coating of MgO. Comparing the photo-thermal composite coatings studied by Liu et al., the present study found out that the MgO coating prepared by changing the current parameters had different heating curves after irradiation under an 808 nm NIR (0.6 W·cm^−2^) light. MAO has some advantages, such as simplicity, high efficiency, and environmental friendliness, and has been diffusely applied to improve the properties of aluminum alloys [22], magnesium alloys [23], and titanium alloys [24]. A MAO coating of Al alloys is mainly used in industry, and the formation of Al_2_O_3_ coating can improve the corrosion resistance of the alloy. A Mg alloy and aTi alloy can be prepared by MAO coating to add the elements required for the human body. Calcium phosphate (Ca-P) coatings fabricated by MAO on AZ31B magnesium improved both the corrosion resistance and the biocompatibility of anodic coating [23]. In order to achieve surface layer biofunctionalization, the MAO coatings doped with Ga ions were fabricated on Ti-6Al-4V alloys [24]. However, the researchers rarely pay attention to the photo-thermal antibacterial property of the component of the MAO coating itself.

Therefore, the present study analyzed the coating morphology, structure, corrosion and photo-thermal property through fabricating MAO coatings on a Mg-3.0Zn-0.2Ca alloy under different current densities and discussed the photo-thermal antibacterial property, as well as the coating photo-thermal mechanism.

## 2. Result

### 2.1. Composition and Surface Morphology of Coated Samples

**Figure 1:**
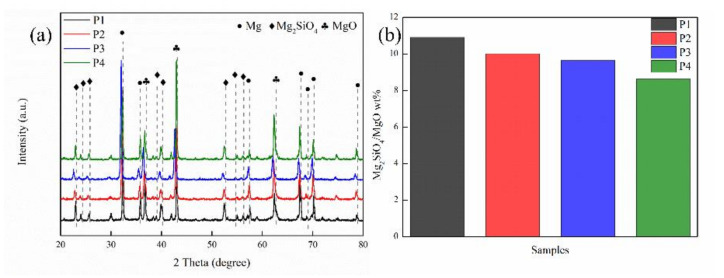
(**a**) XRD patterns of MAO coatings with different current densities; (**b**) histogram of Mg_2_SiO_4_/MgO wt%.

The constitution of arcs during the MAO process could result in the ionization of electrolyte components and the melting of the partial metal surface [25]. The anions in the electrolyte would move towards to the anode under an electric field and reacted with the matrix [26]. Figure 1a below shows the XRD patterns of MAO coatings at four current densities. The characteristic peaks of Mg, MgO, and Mg_2_SiO_4_ appear in all samples. These phases were derived from the magnesium matrix and MAO electrolytes. The Mg phase was spotted due to the X-ray reaching the substrate. The diffraction peaks of MgO are 36.94°, 42.92°, and 62.30°, which respectively respond to the (111), (200), and (220) [16]. The diffraction peaks of the phosphorus phase are not shown in the XRD pattern of the MAO coating, which indicates that compounds containing phosphorus exist in the amorphous state [27]. The jade 6.5 was used to calculate the relative weight percentage of MgO and Mg_2_SiO_4_ of the coating, with its result shown in Figure 1b. The Mg_2_SiO_4_/MgO wt% in MAO coating decreases successively from 10.9 to 10.0, 9.64, and 8.62, respectively, indicating that relative content of MgO in the MAO coating increases gradually with the increase of current density in that the increasing of current density results in the rise of both the number and energy of sparks on the coating surface, and more heat will be generated at the moment of spark discharge, thus promoting the accumulation of MgO wt% in the coating [28]. 

**Figure 2:**
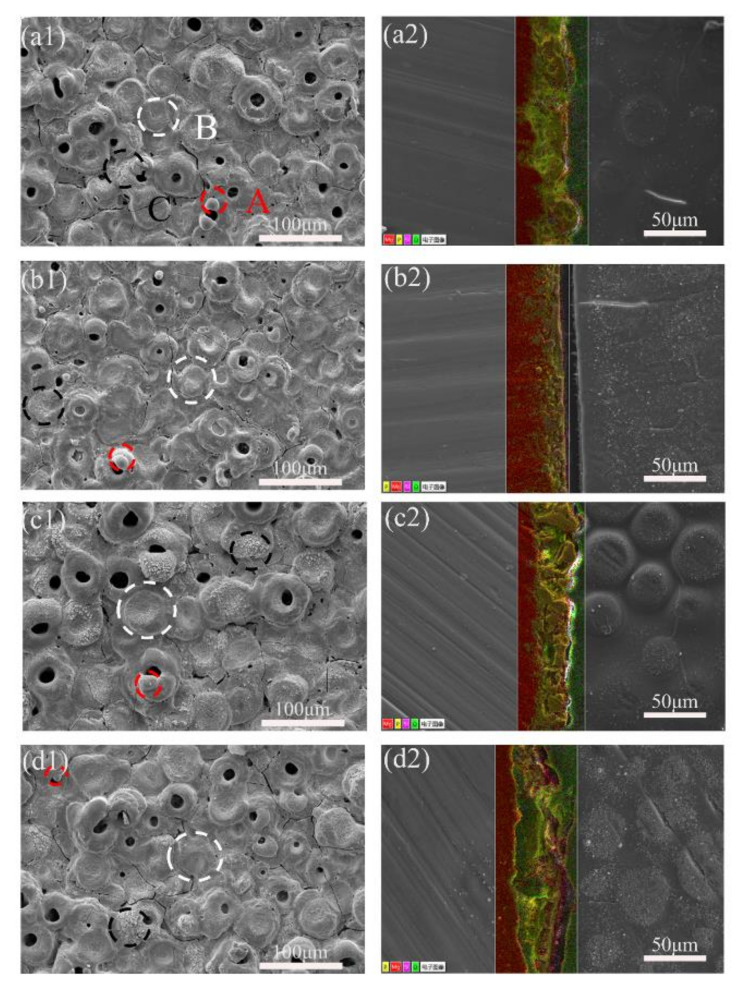
Surface morphology: (**a1**) P1, (**b1**) P2, (**c1**) P3, (**d1**) P4; and cross-sectional EDS of samples with different current densities: (**a2**) P1, (**b2**) P2, (**c2**) P3, (**d2**) P4. (Color code: yellow = P, red = Mg, violet = Si, green = O).

Figure 2(a1–d1) shows the microstructures of the surface of the MAO coating. All of the samples showed typical MAO porous structures with many micro-cracks. The surface of the MAO coatings had three types of micro-pores. A was a dome-shaped protrusion. B was pancake-shaped with an obvious depression in the middle and exhibited a compact structure. C was foam-like and microporous and showed a porous structure [29]. The A, B, and C morphology was formed by A-, B-, and C-type discharge events, respectively [30]. Since the whole MAO process was dominated by B-type discharge, when the current density was increased from 10 A·dm^−2^ to 30 A·dm^−2^, B-type discharge was increased in the MAO process, a denser coating structure with more of the pancake morphology appearing. Therefore, from P1 to P3, the coating density increased; the coating surface crack was relatively lower, and the porosity decreased continuously. But, when the current density was further increased to 40 A·dm^−2^, the positive voltage become higher, while the discharges became acuter on the surface. Although the B-type discharge continued, the MAO coating formed earlier may be melted again, resulting in many micro-pores and micro-cracks on the surface of the P4 coating, and the porosity and roughness increased [31]. ImageJ software was used to calculate the porosity of samples, and it was found that, with the increase of the current density, the change in the porosity of the coating was P1 (4.35%) > P2 (3.29%) > P4 (3.07%) > P3 (2.91%). The results showed that the proper current density could reduce the pore cracks on the surface of the coating and form a smooth and compact coating.

**Figure 3:**
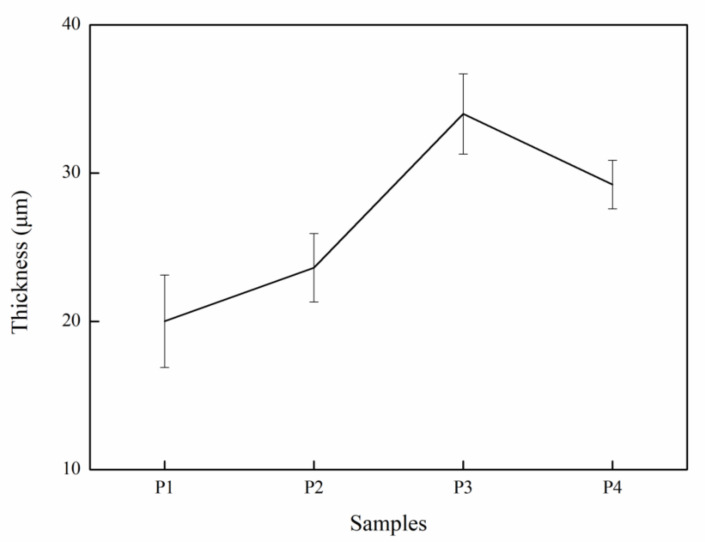
The thickness of MAO coating.

Figure 2(a2–d2) display MAO coatings’ cross-sectional morphologies and element distribution. The structure of the outer layer of the MAO coatings is relatively loose with plenty of isolated pores and through-pores/micro-cracks or pin-holes, while that of the inner layer is dense. Some micro-cracks in the coating were connected to isolated pores [32]. These pores are distributed broadly and randomly in P1, and, compared to that in P1, with the increase of current density, the number of pores in P2, P3, and P4 ranks as P2 > P4 > P3. Figure 3 shows the cross-sectional thickness of the samples. The current density is within a specific range, and the film thickness has a linear relationship with the current density [33]. As the current density increases, the voltage increases, which can effectively improve the energy of plasma discharge. This energy create a high-temperature environment, which makes more metal matrices melt and spray onto the substrate surface, thereby promoting the growth of the MAO coating [28]. The heat generated by the high current density in P4, which has the highest current density, was too much to disperse easily, which led to the deterioration and the dissolution of the MAO coating [34], making the pores of the P4 increase again. When the density of the MAO coating decreases, corrosive ions and the water penetration rate into the coating will be accelerated, thus reducing its corrosion resistance [35]. In conclusion, when the current density was 30 A·dm^−2^, the sample had the lowest surface porosity, a uniform cross-section, and a compact structure.

### 2.2. Corrosion Resistance of the MAO Coating

**Figure 4:**
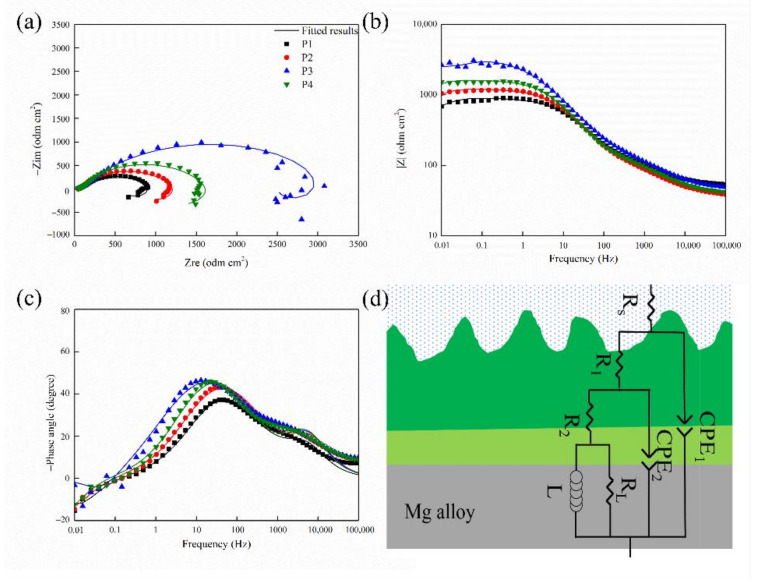
(**a**) Nyquist, (**b**) Bode, and (**c**) Bode phase angle plots and (**d**) equivalent circuit models of all samples.

The EIS results of the different MAO coatings are demonstrated in Figure 4. Nyquist plots of the samples are presented in Figure 4a. All four MAO coatings display an inductive loop and two capacitance loops in the SBF solution. However, the Nyquist plots of the samples show a significant difference in ring size with different current densities; moreover, the larger capacitance loop, the better the anti-corrosion effect. The anti-corrosion of the samples could be displayed in increasing order as follows: P1 < P2 < P4 < P3, confirming that the MAO coating protected the Mg substrate from corrosion [36].

Under normal circumstances, a high modulus |Z| value suggested that the sample had better anti-corrosion [37]. The |Z| of the samples could be displayed in increasing order as follows: P1 < P2 < P4 < P3. This result indicates that MAO improved the corrosion resistance of the samples. 

Moreover, the MAO coating exhibited two-time constants corresponding to the Bode phase plot’s multi-layer structure (Figure 4c). The phase angle of the intermediate frequency range became smoother, which indicated that the coating was denser [32].

The corresponding equivalent circuits (ECs) of the MAO coating are shown in Figure 4d to illustrate the corrosion resistance of the samples further. All samples were fitted with an equivalent circuit diagram, and the corresponding fitting data are listed in Table 1. Rs represents the solution resistance. R1 and R2 stand for resistors of the outer and inner layers, respectively. Inductance (L) refers to the pitting that occurred in the low-frequency region [38]. CPE1 and CPE2 represent the constant-phase elements in the outer and inner layers. The n value is the dispersion-effect index of CPE. The R1 value of the impedance element of a loose layer of the MAO film was relatively low. The large diameter of micro-pores on the loose layer and the infiltration of the SBF solution into the loose layer result in a small impedance modulus. The R2 represents the impedance element in the inner layer of the MAO film. As what shows in the list, the value of R2 is high. Because the inner discharge channel size was small, its impedance value was enormous [39].

The corrosion resistance of the MAO film is related to its composition and compactness. The MgO affects the corrosion resistance of the coating. As the current density was increased from 10 A·cm^−2^ to 30 A·cm^−2^, the content of MgO increased, the size of the coating surface micro-pores decreased, and the thickness of the film increased. Therefore, the corrosion-resistance performance improved [40]. However, when the current density rose to 40 A·cm^−2^, the degree of melting of Mg^2+^ increased, creating more MgO. However, compared with sample P3, the corrosion resistance of P4 was reduced due to the micro-pores and micro-cracks on its surface caused by relatively higher current density.

The MAO coatings fabricated under different current densities on the surface of the Mg alloy could efficiently improve the anti-corrosion property of the Mg alloy. The excellence of the anti-corrosion performance resulted from different current densities can be displayed as 30 A·dm^−^^2^ > 40 A·dm^−2^ > 20 A·dm^−^^2^ > 10 A·dm^−2^. Therefore, the corrosion resistance of sample P3 prepared by current density 30 A·cm^−2^ was the best.

**Figure 5:**
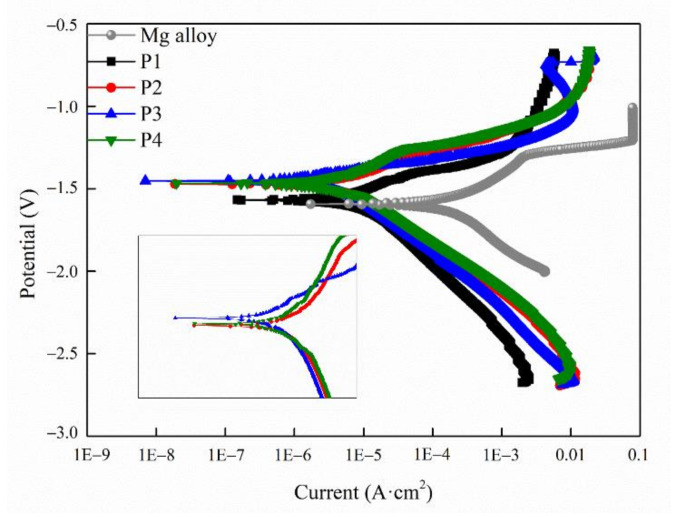
Potentiodynamic polarization curves of the different samples tested in SBF.

Figure 5 corresponds to the potentiodynamic polarization curves of the MAO coating. *β_a_* and *β_c_*, respectively, represent the polarization slope of the anode and cathode; *R_p_* represents the polarization resistance, and *E_corr_* and *I_corr_* refer to the self-etching potential and self-etching current density, respectively. The fitting results was reached by the Tafel extrapolation method and is displayed in Table 2. The more positive the *E_corr_* and lower the *I_corr_* of the coating, the more stable the coating would be [41]. Compared with the relevant results, it can be seen that the self-corrosion potential of P3 reaches up to −1.45 V and that its self-corrosion current density is the lowest as 1.08 A × 10^−6^ A·cm^2^, indicating that P3 had good corrosion resistance, which is consistent with the EIS results.

**Figure 6:**
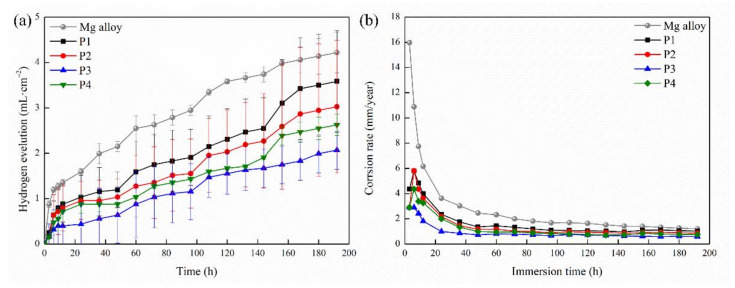
(**a**) HE curves and (**b**) corrosion rate images of the samples immersed in SBF solution for 192 h.

The H_2_ release volume of all samples in SBF is shown in Figure 6a. The variation trend of the hydrogen release volume of all samples was similar with the increase in soaking time, as the hydrogen release volume of all samples increased rapidly with the initial soaking stage, and then the increase slowed down [42]. In comparison, the amount of the hydrogen release of the uncoated Mg alloy soaked in SBF for 12 h was significantly more than twice that of the MAO-coated sample. Then the amount of hydrogen release decreased, but the amount of the hydrogen release of the Mg alloy was the highest during soaking. The results demonstrated the protective effect of the MAO coating on the magnesium alloy. Through the comparison of hydrogen evolution curves of layers with different current densities, the P3 samples showed the best corrosion-resistance performance. The amount of hydrogen evolution at 48 h was only 1/3 that of the bare alloy and 1/2 that of the P4 sample, significantly lower than that of the P1 and P2 samples. The amount of hydrogen evolution after soaking for 196 h consistently increased at a relatively slow rate. However, the hydrogen evolution amount of P1, P2, and P4 samples increased significantly after being soaked for 160 h. The results indicated that local rupture appeared on the coating surface, weakening its protective effect. Compared with that of the P1, P2, and P4 samples, the coating of the P3 sample had a smaller hydrogen evolution amount, illustrating that the P3 sample possessed the most stabilized anti-corrosion.

As shown in Figure 6b, the corrosion rate of all of the samples decrease with the soaking time, and the corrosion rate of the coated alloy is significantly lower than that of the bare alloy with the same soaking time. After being soaked for 196 h, the corrosion rate of the Mg alloy was 1.2021 mm·year^−1^ and that of samples P1, P2, P3, and P4 were 1.02065 mm·year^−1^, 0.86188 mm·year^−1^, 0.58971 mm·year^−1^, and 0.74848 mm·year^−1^, respectively. In general, the corrosion rate of the MAO coating was lower than that of the Mg substrate. Among them, the P3 sample had the lowest annual corrosion rate. 

### 2.3. Photo-Thermal Effect and Mechanism of MAO Coatings with Different Current Densities

**Figure 7:**
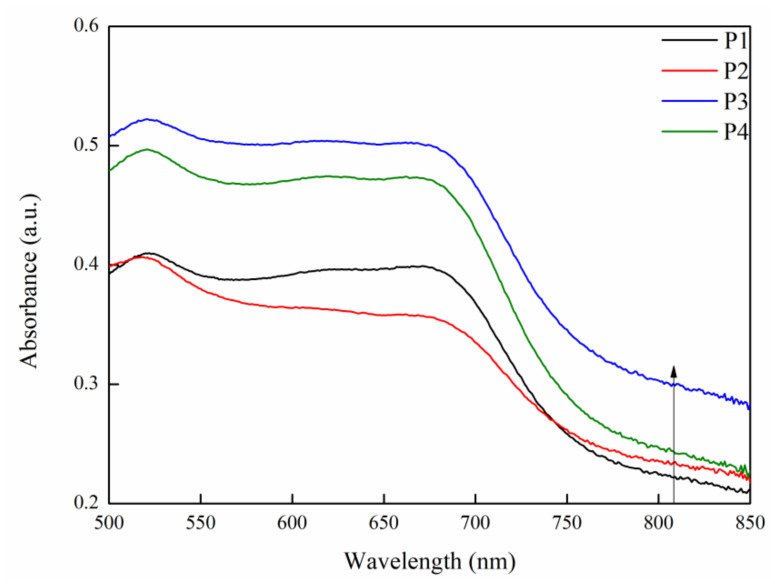
The UV–vis spectra of P1, P2, P3, and P4.

The UV–vis was used to verify the light absorption capacity of the samples, with its results shown in Figure 7. It can be seen that, at 808 nm, with the increase of current density from 10 A·dm^−2^ and 20 A·dm^−2^ to 30 A·dm^−2^, the light absorbance of the MAO coatings keep rising, while, when it reaches 40 A·dm^−2^, the light absorbance declines. In other words, the light absorption capacity of P3 was the finest. According to the XRD analysis of the samples (Figure 1), the content of MgO in the coating increases with the increase of current density in the MAO process, and MgO can promote charge separation [16]. Through density functional theory calculations, N. Pathak’s study showed that introducing oxygen vacancies states near the conduction band was an efficient way to reduce the optical band gap. In the composition and structure of the MAO coating on a magnesium alloy, MgO has an excellent photocatalytic performance by introducing defects such as oxygen vacancy [40]. As the current density increases further, the content of MgO reaches the highest in the P4 sample, but its absorption intensity at 808 nm is lower than that of the P3 sample, as shown in Figure 7. The results show that excessive MgO would hinder the light absorption performance and reduce the absorption strength of the sample. The reason may be that excessive MgO increased the electron-hole recombination rate in the coating [43].

To detect the photo-thermal properties of the MAO coating under 808 nm (1.0 W·cm^−2^) laser irradiation, the photo-thermal images were procured, and the photo-thermal heating curves were drawn. 

**Figure 8:**
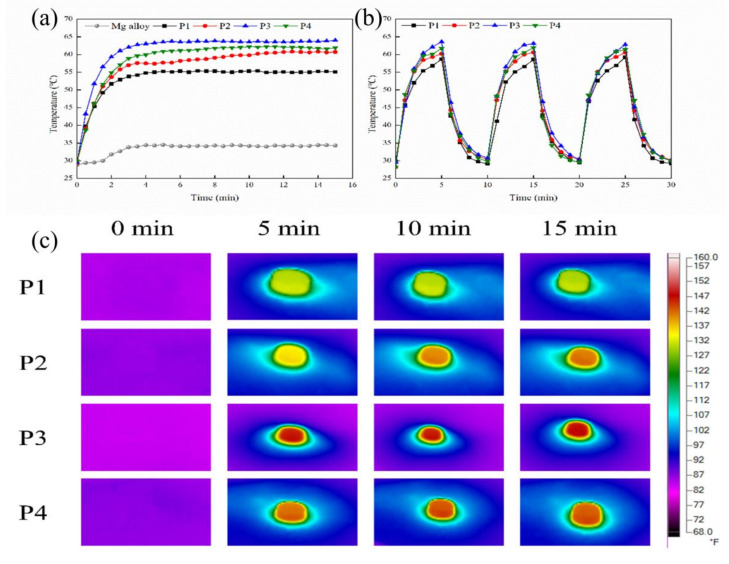
The characterization of the photo-thermal properties of the different samples: (**a**) heating curves of Mg, P1, P2, P3, and P4 (1.0 W/cm^2^, 15 min); (**b**) the heating and cooling curves of the different samples for three cycles; (**c**) the real-time pictures taken during the light irradiation process of different samples, (*F: Fahrenheit degree).

According to Figure 8a, after being shined with an 808 nm NIR laser for 15 min, the ultimate temperatures of the Mg and MAO coatings were approximately 34.3 °C, 55.1 °C, 60.7 °C, 64 °C, and 61.9 °C respectively. The temperature rise of the P3 coating is the most obvious. The photo-thermal performance was superior, and its maximum temperature exceeded that of other MAO coatings. The results show that the coating with a current density of 30 A·dm^−2^ had the most excellent photo-thermal property.

As shown in Figure 8b, the temperature with NIR 808 nm laser irradiation stays steady after three periods, showing that all of the MAO coatings possessed a steady photo-thermal effect. Figure 5c shows the real-time infrared thermal pictures of the different samples during the 808 nm NIR laser irradiation. The incremental sequence of the calefaction rates of the samples is as follows: P3 > P4 > P2 > P1 > Mg. 

The results showed that the coating exhibited excellent photo-thermal performance under NIR 808 nm laser irradiation [44,45].

Previous studies have shown that oxygen vacancy (OV) was a metal oxide defect caused by a specific external environment (such as high temperature, etc.) [46]. It made the oxygen leave the lattice node. OVs can improve photocatalytic activity by capturing electrons as shallow electron traps and significantly improve the photo-thermal effect of the catalyst [47]. The process of the plasma discharge of micro-arc oxidation involved the fluctuation of electric parameters, high temperature, high pressure, oxygen generation, local oxide crystallization, and the internal stress of the oxide layer. Thus, the MgO generated in the coating had a photocatalytic performance.

Therefore, P3 with the highest temperature was taken as an example for the characterization of oxygen vacancies.

**Figure 9:**
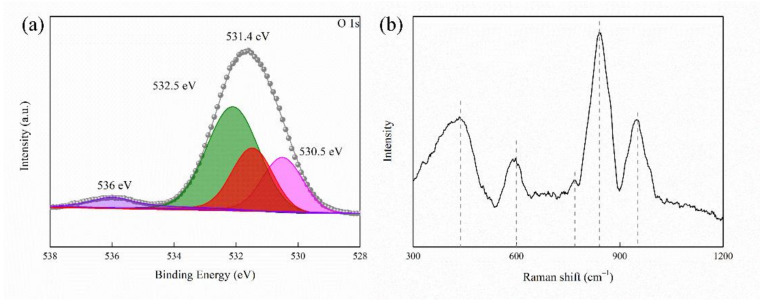
(**a**) XPS high-resolution O 1 s spectra of P3 and (**b**) Raman spectra of P3.

Firstly, the sample was studied by XPS, with its result shown in Figure 9a. The peak at 530.5 eV stands for lattice oxygen. The peak at 532.5 eV represents adsorbed oxygen. The peak at 531.4 eV refers to oxygen vacancies [45]. The peak at 536 eV is the gas phase CO_2_ [47].

Furthermore, Figure 9b shows the Raman spectroscopy of the samples, further unveiling the existence of oxygen vacancies in the structure of the MAO coating. The band at 450 cm^−1^ represents the vibration of MgO, and the peak at 769 cm^−1^ is assigned to Mg−O-Si bonds. In addition, the band at 600 cm^−1^ refers to oxygen vacancies [48]. The asymmetric stretching between the phosphate and hydroxyl group can be seen at 850 cm^−1^. The peak around 952 cm^−1^ can be shown with a symmetric extension between P and O atoms.

Combined with the Raman and XPS characterizations, the assumption can be made that the oxygen vacancies of P3 were subsistent. 

**Figure 10:**
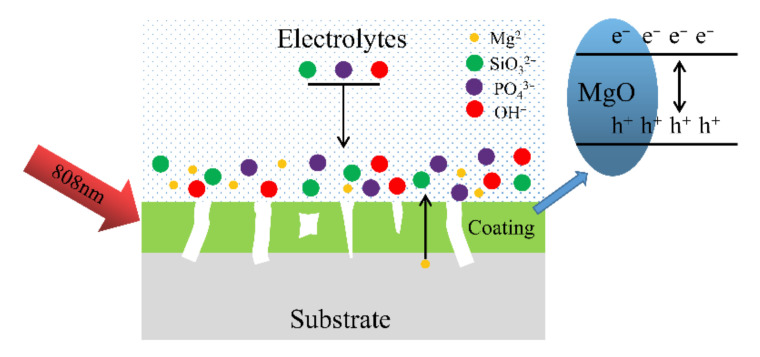
Schematic illustrating the mechanism of the photocatalytic property of the MAO coating.

Based on the research results above, the photocatalytic mechanism of the coating is shown in Figure 10. MAO is a technology mainly composed of matrix element oxides on the surface of the matrix metal. In the initial stage of the MAO process, an anodic oxide layer is formed on the substrate surface as a barrier layer, and electrons are continuously transfered from the electrolyte into the barrier layer. Due to the structural defects in the barrier layer, when the voltage reaches the critical voltage of the barrier layer, the electron avalanche effect might occur in the barrier layer under the reaction of a strong electric avalanche [49] and further spark an anodic oxidation reaction, and a micro-arc oxidation reaction will appear. Finally, the MAO coating is formed. The MAO coating had an absorption strength at 808 nm, indicating that luminescent electron carriers could be generated on the sample’s surface. Oxygen vacancies promote the capture and migration of pores [47]. MgO promotes the separation of photo-generated electron carriers [44] and also hinders the recombination of photo-generated electron carriers and holes. The longer the separation time of the photoelectron carrier and hole, the stronger the photocatalytic ability. Therefore, the MAO coating had an excellent photo-thermal ability.

### 2.4. The Antibacterial Mechanism of the MAO Coating

**Figure 11:**
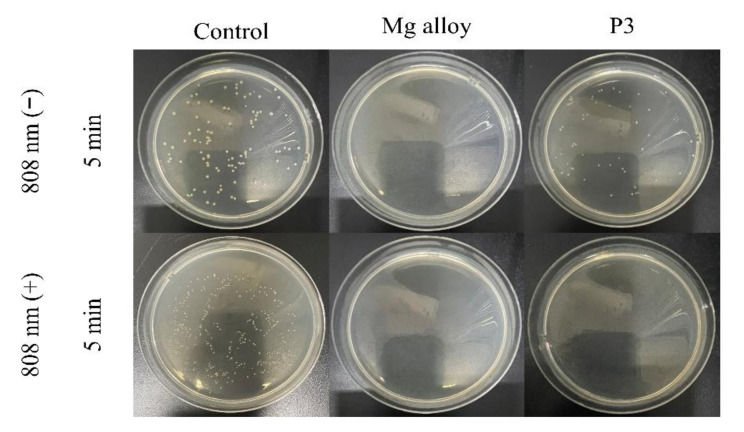
The antibacterial properties of samples against *S. aureus*.

**Figure 12:**
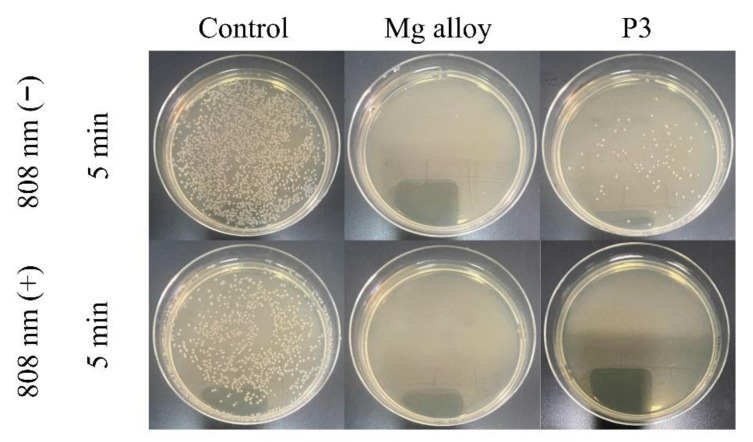
The antibacterial properties of samples against *E. coil*.

The plate-counting method has been used to assess the antibacterial efficacies of the bare and coated Mg alloy against *S. aureus* and *E. coil* bacteria. The result is shown in Figure 11 and Figure 12. The viable colonies of *S. aureus* and *E. coil* grow well on LB agar plates when the bacteria are cultured without samples (control). There is no significant effect on the survival rate of bacteria under 808 nm light (control under light), which shows that the 808 nm light alone did slight damage to bacteria. Compared with the control group, the P3-coated samples showed only weak antibacterial ability under the condition of no light. However, the bare Mg alloy almost entirely killed off bacteria, and its antibacterial activity was significantly better than that of the MAO coating. When the samples and bacteria could be cultured together, the bare Mg alloy would quickly be corroded and produce OH^−^, making the pH value of the solution as high as 9.5. Previous studies have shown that the antibacterial activity could be improved by increasing the pH value from 7.4 to 10. The antibacterial property of the bare Mg alloy was the rapid increase of the pH value of the surrounding solution caused by its degradation. However, these conditions were also not conducive to the growth and reproduction of normal cells [50]. After irradiation with an 808 nm laser for 5 min, the MAO coating of sample P3 showed the same antibacterial property as the bare Mg alloy. The viable colonies of *S. aureus* and *E. coil* were almost completely gone. The pH value of the solution of the sample was about 8.4. It could be seen that it was the photo-thermal effect with light treatment, rather than the change in the pH value, that contributed to the good antibacterial activity of sample P3.

**Figure 13:**
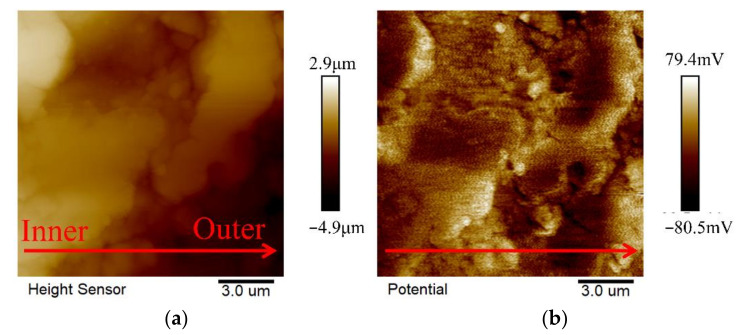
MAO cross-section AFM morphology and potential diagram of P3.

In order to explore the antibacterial mechanism of the MAO-coated sample, AFM was used to test the height difference and the potential distribution of the P3 cross-section from inside to outside, and the result is shown in Figure 13. The cross-section of the MAO coating was relatively flat, and the maximum height difference between the inner and outer layers was about 5 μm, as shown in Figure 13a. The outer layer potential of the corresponding MAO coating was also slightly lower than the inner layer, with a potential difference of about 100 mV, as shown in Figure 13b. Therefore, when the MAO coating was put into the bacterial solution, with the continuous infiltration of the solution into the coating, micro galvanic corrosion would occur between the inner layer and the outer layer due to different chemical potentials. However, the intensity of galvanic corrosion was much lower than that of the degradation of the bare Mg alloy. Therefore, in the same contact time, the change in the pH value of the bacterial solution also significantly decreased and only partially killed the bacteria.

**Figure 14:**
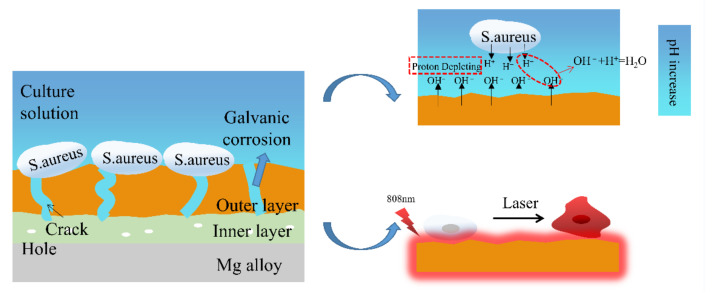
The antibacterial mechanism of MAO coating under photo-thermal action.

The experimental data analysis mentioned above shows the antibacterial mechanism of the MAO coating on the Mg alloy surface in Figure 14. Firstly, galvanic corrosion occurred because of the electric potential difference between the inside and the outside of the MAO coating, so the MAO-samples surfaces could continuously release OH^−^. The released OH^−^ would constantly react with bacterial extracellular H^+^. The continuous consumption of H^+^ caused by the reaction formed an H^+^ consumption transition zone. Since an appropriate concentration of H^+^ was a necessary condition for the energy-dependent activities of bacteria, the excessive consumption of H^+^ would destroy the normal functions of the bacteria and eventually result in a defective development or final death [51]. Secondly, through thermal treatment with 808 nm light, a local high temperature appeared on the surface of the sample coating, and bacteria damage such as protein/enzyme denaturation, cell membrane rupture, cell cavitation, and cell fluid evaporation happened and led to the ultimate killing of bacteria [44,45].

Therefore, the MAO coating of sample P3 showed excellent antibacterial property.

## 3. Materials and Methods

### 3.1. Materials and MAO Coatings Preparation

The as-extruded Mg-3Zn-0.2Ca alloy, cut into a cylinder with a size of ϕ 8 cm × 3 cm (mass fraction: Zn 3.0%, Ca 0.2%, balance Mg), was used as materials. For MAO process, all samples were ground by silicon carbide (SiC) paper progressively up to 3000#, then cleaned in ethanol and DI water for 5 min respectively, and finally dried in air.

The MAO coatings were prepared on equipment comprising an MAO-50D power supply (Tongchuang Technology Co., Ltd., Chengdu, China). The electrolyte was a hybrid of 10 g/L Na_2_SiO_3_, 5 g/L NaOH, and 3 g/L (NaPO_3_)_6_ (all drugs source from Aladdin Co., Ltd., Shanghai, China). The samples were taken as anode, and stainless steel was used as the cathode. The mode of MAO was constant current. Frequency, positive duty cycle, negative duty cycle, and oxidation time were fixed at 500 Hz, 40%, 20%, and 12 min, while the current densities were 10, 20, 30, and 40 A·dm^−2^, respectively. The samples were named P1, P2, P3, and P4, respectively.

### 3.2. Characterization of the Coatings

The crystallographic structure was spotted through an X-ray diffractometer (XRD, Rigaku D/max-2500, Tokyo, Japan) with a Cu target (λ = 0.154 nm) from 20° to 80° at a scanning rate of 5° min^−1^. Jade 6 was used to analyze the relative content of the phase in the XRD pattern. The field-emission scanning electron microscopy (SEM, Quanta FEG 250, Ann Arbor, MI, USA) was used to observe the MAO coatings’ surface and cross-sectional morphologies. Elemental compositions were analyzed by an SEM additional energy-dispersive X-ray spectrometry (EDS) device. A UV–Vis spectrometer (Lambda 750 Perkin Elmer, Norwalk, CT, USA) was used to examine the absorption spectra of the samples. X-ray photoelectron spectroscopy (XPS) and Raman spectroscopy (ESCALAB 250 xI Thermo Fisher Scientific, Waltham, MA, USA) were used to identify the oxygen vacancies on the surface. An atomic force microscope (AFM, Dimension icon, Billerica, MA, USA) was applied to analyze the potential distribution on the cross-section of the MAO coating.

### 3.3. Photo-Thermal Property Measurement

The photo-thermal property of the samples radiated with an 808 nm NIR laser was gauged and reported by an infrared imaging device (TIS 75+, Shanghai, China). The five groups (Mg, P1, P2, P3, and P4) were irradiated by an 808 nm NIR laser for 15 min (1.0 W·cm^−2^). The infrared imaging device procured the infrared thermal pictures every 5 min. 

### 3.4. Corrosion Characterization

The anti-corrosion of the samples were evaluated via potentiodynamic polarization curves (PDP) and electrochemical impedance spectroscopy (EIS) using an electrochemical device (zennium, Zahner, Germany). A three-electrode cell with the samples was taken as the working electrode with an exposed area of 1 cm^2^; a saturated calomel electrode (SCE) was taken as the reference electrode, and an electrode was taken as the counter electrode. All electrochemical surveys were conducted in a SBF solution (6.5453 g/L NaCl, 2.2683 g/L Na_2_CO_3_, 0.3728 g/L KCl, 0.1420 g/L Na_2_HPO_4_, 0.3050 g/L MgCl_2_·6H_2_O, 0.2780 g/L CaCl_2_, 0.0711 g/L Na_2_SO_4_, 6.057 g/L tris) at 37 °C. The PDP was obtained at a scan rate of 1 mV/s. In EIS, the frequency range was set as 10^5^ Hz to 10^−2^ Hz. The polarization resistance (*R_p_*) was calculated using the Equation (1):

(1) $$R_{P}=\frac{\beta_{c}\times \beta_{a}}{2.303\times \left(\beta_{c}+\beta_{a}\right)\times I_{corr}}$$

The hydrogen evolution test was conducted to evaluate the long-term corrosion behavior of the samples immersed in a SBF solution at 37.5 °C for 192 h. The hydrogen evolution rate VH of the sample was calculated according to the volume of hydrogen precipitation, and then the corrosion rate PH was calculated according to Equation (2).

(2) $$P_{H}=2.2779V_{H}$$

### 3.5. In Vitro Antibacterial Test

The frozen solution of *S. aureus* and *E. coli* were placed in a 37 °C water bath for rapid thawing; 100 μL was transferred to a fresh broth medium and cultured in a 37 °C (170 r/min) constant-temperature oscillator for 18 h. Then, a 100 μL bacterial solution was placed in a test tube with a 1 mL 0.9 wt% NaCl solution, and then different samples were added and placed in a constant-temperature shaker at 37 °C for 2 h. The experimental laser group was continuously irradiated with an 808 nm NIR (1.0 W·cm^2^) for 5 min, and then all *S. aureus* and *E. coil* suspensions in the control group and the experimental laser group were diluted by 10^6^ times. A total of 100 μL diluted bacterial suspension droplets were placed on a nutrient agar plate, coated evenly with a coating rod, and cultured in a constant-temperature incubator at 37 °C overnight.

## 4. Conclusions

The conclusions of the present study are as follows:

(1) The MAO coating fabricated at the current density of 30 A·dm^−2^ possessed the best structure with fewer defects and thicker thickness.

(2) The MAO coatings fabricated under different current densities on the surface of the Mg alloy could efficiently improve the anti-corrosion property of the Mg alloy. The excellence in anti-corrosion performance resulting from different current densities can be displayed as 30 A·dm^−2^ > 40 A·dm^−2^ > 20 A·dm^−2^ > 10 A·dm^−2^.

(3) When the current density was changed from 10 A·dm^−2^ to 30 A·dm^−2^ on the fabricated MAO coating, the content of MgO in the coating kept rising with the increase of current density. The 808 nm absorption intensity increased. When the current density was increased to 40 A·dm^−2^, the 808 nm absorption intensity declined. The excellence in photo-thermal performance resulting from different current densities can be displayed as 30 A· dm^−2^ > 40 A·dm^−2^ > 20 A·dm^−2^ > 10 A·dm^−2^.

In conclusion, below 30 A·dm^−^^2^, with the increase of current densities set to the MAO coatings, the corrosion resistance and photo-thermal property of the sample coating kept increasing continuously, with the optimal performance reached at 30 A·dm^−^^2^.

## Figures and Tables

**Table 1:** Electrochemical data obtained from the equivalent circuit fitting of EIS curves.

Sample	R_s_ (ohm·cm^2^)	CPE_1_ (μF·cm^−2^)	n_1_	R_1_ (ohm·cm^2^)	CPE_2_ (μF·cm^−2^)	n_2_	R_2_ (ohm·cm^2^)	R_L_ (ohm·cm^2^)	L (H·cm^−2^)
P1	51.42	2.88 × 10^−5^	0.68	111.80	2.99 × 10^−5^	0.80	416.10	323.60	3.68 × 10^3^
P2	36.24	3.36 × 10^−5^	0.66	111.90	2.62 × 10^−5^	0.81	574.10	494.80	8.59 × 10^3^
P3	51.41	3.57 × 10^−5^	0.62	218.50	2.90 × 10^−5^	0.78	2654.00	776.00	2.19 × 10^3^
P4	39.26	2.65 × 10^−5^	0.67	122.20	2.85 × 10^−5^	0.83	688.60	771.50	2.06 × 10^4^

**Table 2:** Electrochemical data obtained via the Tafel fitting of PDP curves.

Sample	*I_corr_* (A/cm^2^)	*E_corr_* (V)	*β_a_* (mV)	*−β_c_* (mV)	*R_p_* (Ω·cm^2^)
Mg alloy	3.52 × 10^−5^	−1.59	333.18	607.74	0.26 × 10^3^
P1	5.47 × 10^−6^	−1.57	166.59	203.68	5.88 × 10^3^
P2	5.44 × 10^−6^	−1.47	181.61	294.38	8.30 × 10^3^
P3	1.08 × 10^−6^	−1.45	72.47	133.75	1.70 × 10^4^
P4	4.21 × 10^−6^	−1.47	206.03	218.87	1.13 × 10^4^

## Data Availability

Not applicable.
